# General Health Benefits and Pharmacological Activities of *Triticum aestivum* L.

**DOI:** 10.3390/molecules27061948

**Published:** 2022-03-17

**Authors:** Said Moshawih, Rabi’atul Nur Amalia Abdullah Juperi, Ganesh Sritheran Paneerselvam, Long Chiau Ming, Kai Bin Liew, Bey Hing Goh, Yaser Mohammed Al-Worafi, Chee-Yan Choo, Shobna Thuraisingam, Hui Poh Goh, Nurolaini Kifli

**Affiliations:** 1 PAP Rashidah Sa’adatul Bolkiah Institute of Health Sciences, Universiti Brunei Darussalam, Gadong BE 1410, Brunei; saeedmomo@hotmail.com (S.M.); 19b3031@ubd.edu.bn (R.N.A.A.J.); pohhui.goh@ubd.edu.bn (H.P.G.); 2 School of Pharmacy, Taylor’s University, Subang Jaya 47500, Malaysia; ganesh_alei@hotmail.com; 3 Faculty of Pharmacy, University of Cyberjaya, Cyberjaya 63000, Malaysia; liewkaibin@cyberjaya.edu.my; 4 Biofunctional Molecule Exploratory Research Group (BMEX), School of Pharmacy, Monash University Malaysia, Bandar Sunway 47500, Malaysia; goh.bey.hing@monash.edu; 5 College of Pharmaceutical Sciences, Zhejiang University, Hangzhou 310058, China; 6 College of Medical Sciences, Azal University for Human Development, Amran P.O. Box 447, Yemen; yworafi@yahoo.com; 7 College of Pharmacy, University of Science and Technology of Fujairah, Fujairah P.O. Box 2202, United Arab Emirates; 8 Faculty of Pharmacy, Universiti Teknologi MARA, Puncak Alam 42300, Malaysia; choo715@uitm.edu.my; 9 Faculty of Science, Universiti Putra Malaysia, Seri Kembangan 43400, Malaysia; shobnasima@gmail.com

**Keywords:** wheat, *Triticum aestivum*, functional food, anticancer, antimicrobial, antidiabetic, laxative

## Abstract

Common wheat (*Triticum aestivum*), one of the world’s most consumed cereal grains, is known for its uses in baking and cooking in addition to its medicinal uses. As this plant’s medical benefits are enormous and scattered, this narrative review was aimed at describing the pharmacological activities, phytochemistry, and the nutritional values of *Triticum aestivum*. It is a good source of dietary fiber, resistant starch, phenolic acids, alkylresorcinols, lignans, and diverse antioxidant compounds such as carotenoids, tocopherols and tocotrienols. These constituents provide *Triticum aestivum* with a wide range of pharmacological properties, including anticancer, antimicrobial, antidiabetic, hypolipemic, antioxidant, laxative, and moisturizing effects. This review summarized the established benefits of wheat in human health, the mode of action, and different clinical, in vitro and in vivo studies for different varieties and cultivars. This review also gives an insight for future research into the better use of this plant as a functional food. More clinical trials, in vivo and in vitro studies are warranted to broaden the knowledge about the effect of *Triticum aestivum* on nutrition-related diseases prevention, and physical and mental well-being sustenance.

## 1. Introduction

Wheat is one of the world’s most consumed cereal grains, along with rice and maize. The wide range of varieties of wheat contributed to its popularity. The top wheat producer was China, as it produced around 30% (112 to 120 million tons) [1] of the overall world wheat production between 2012 and 2016. Other countries that accounted for 63.46% of the world wheat production are India, the Russian Federation, the United States of America, and Canada [2]. Wheat is commonly used in cooking and baking. However, it was discovered that different parts of the wheat plant have medicinal uses such as wheat bran with anticancer properties and wheat sprouts for their antimicrobial activities [3].

Wheat is an excellent energy source as it contains carbohydrates and significant amounts of other crucial nutrients such as proteins, dietary fiber, and smaller amounts of lipids, terpenoids, vitamins, minerals, and phytochemicals [4]. According to the Healthy U.S. Style Eating Pattern, 2000 calories per day is six ounce-equivalents daily, half of which should be whole grains [5]. Whole grains are great sources of nutrients, such as dietary fiber, iron, zinc, manganese, folate, magnesium, copper, thiamin, niacin, vitamin B6, phosphorus, selenium, riboflavin, and vitamin A. Studies done by Liu et al. [6], Meyer et al. [7], and Parker et al. [8] supported the statement that consumption of two or more servings of whole grains a day may reduce the risk of cancer, cardiovascular diseases and diabetes mellitus type II. This review aims to comprehensively compile an extensive report on the pharmacological activities, medicinal use, phytochemistry, and the nutritional values of *Triticum aestivum* (T.A.). Moreover, we will further provide an in-depth discussion of T.A. applications in diverse medicinal purposes as a staple and functional food.

## 2. Botanical Description

This annual grass forms either solitary or tufted leafy culms about 2 to 3 and a half inches tall. The culms are light green, erect, terete, glabrous, and sometimes glaucous. Along the length of each culm, alternate leaves grow. The leaf blades are 6–18 mm across and 5–12 inches long. The leaves appear bluish or grayish-green, glabrous, and sometimes glaucous. These blades are ascending, arching, or rather floppy. The bases of these blades often have rounded auricles with scar-like wavy margins. The open leaf sheaths have the same characteristics as the leaves. The ligules are short-membranous and are about 1–2 mm in length; meanwhile, the nodes are swollen and glabrous. Each culm ends in an erect floral spike about 2–4 inches long. These floral spikes are also grayish or bluish-green with additional darker markings. Furthermore, each floral spike has multiple overlapping spikelets appressed against the rachis, which is the central stalk of the spike that is also nearly erect [9].

Each spikelet is about 10–15 mm in length, consisting of a pair of glumes at the base along with 2–5 florets with lemmas above. The glumes are 9–11 mm long and are ovate in shape, partially keeled, and glabrous. The lemmas are around 9–11 mm in length, ovate in shape, convex along their surfaces, and glabrous. Along the inner sides of the florets are membranous paleas similar to the lemmas. At the apex of both the glumes and lemmas, there are 1–2 small teeth. Each floret consists of an ovary, a pair of feathery stigmata, and stamens. The blooming period usually occurs from the late spring to mid-summer. The wind cross-pollinates the florets. They are replaced by grains that are 7.5–8.5 mm long and 3.5–3.75 mm across and ovoid-ellipsoid in shape. The light-colored grains are convex on one side and incurved on the other. The root system is fibrous [9].

For wheat cultivation, the preferred conditions are places with full sun, mesic to dry-mesic conditions, and soil containing loam or clay-loam. Some varieties of wheat, such as winter wheat, are planted during the fall season, while other varieties such as spring wheat are planted during spring. Wheat originated from the eastern part of the Mediterranean or the Middle East in Eurasia and is considered the major agricultural crop. Typical habitats of such plants include fields, roadsides, railroads, areas near grain elevators, and open waste areas. Sometimes T.A. is deliberately planted as a food source for wildlife and to control erosion along roadside embankments until perennial grasses become established. Wheat thrives in highly disturbed areas with exposed topsoil [9]. Dried wheat is typically milled into flour which is then used to create many different flour-based foods such as bread, pasta, cereal, and many more. The structure of wheat grain, the function of each wheat part, and the taxonomical classification of T.A. are shown in Table 1 and Table 2, respectively.

## 3. Phytochemistry of *Triticum aestivum*

Wheat products are divided into three fractions: bran, germ, and endosperm. Ninety percent of the dry weight of mature wheat grain comprises three major components: starch, proteins, and cell wall polysaccharides. Wheat bran is a major byproduct of wheat grain milling when producing white or refined flour. At the same time, the rest are other minor elements, including lipids, terpenoids, phenolics, minerals, and vitamins. Lipids make up about 8–15% of the germ, around 6% of the bran, and 1–2% of the starchy endosperm in the whole wheat kernel. Many vital minerals and antioxidants are included in the germ, as well as natural oil (wheat germ oil). When a germ is crushed and exposed to air, the oil begins to oxidize, eventually becoming rancid. The removal of the germ extends the shelf life of flour and eliminates the risk of rancid oils imparting a strong taste to the flour [13]. Germ lipids are primarily nonpolar (up to 85%), with polar lipids accounting for just a small part (up to 17%), but the profile of endosperm lipids differs significantly from that of germ and bran lipids. Among the various wholegrain components, endosperm lipids are the only significant source of galactolipids (primarily monogalactosyl diglyceride and digalactosyl diglyceride) and phospholipids (primarily phosphatidylcholine, lysophosphatidylcholine, phosphatidylethanolamine, and lysophosphatidylethanolamine) [14]. The general nutrient composition of wheat bran, micronutrients found in wheat bran, and nutrient composition in whole wheat grain flour are shown in Table 3, Table 4 and Table 5, respectively.

Different solvent systems were employed using the Soxhlet apparatus to extract T.A. grass and it was found that they contain rutin, chlorogenic acid, tocopherol, and gallic acid in the organic solvents; hexane, chloroform, and methanol [20]. Those organic extracts showed antimicrobial activity against *Salmonella typhi*, *Staphylococcus aureus*, and *Vibrio cholerae*. The evaluation of carotenoids (lutein and zeaxanthin) and phenolic acid concentrations of the whole wheat in Titlis and Runal cultivars over several years was tested under conventional and organic production methods [21]. Up to 98% of the phenolic acids were bound to cell components such as ferulic acid. Ferulic acid comprises 85% of the total phenolic acids followed by coumaric, sinapic, and caffeic acids. Generally, both the conventional- and organic-produced T.A. yielded similar concentrations of phytochemicals. Marrelli et al. [22] studied two wheat varieties from Italy: Carosella and Majorca. High concentrations of polyphenols and antioxidant activities were found in both varieties and considered as potential sources of antioxidants with IC_50_ values of 0.008 mg/mL and 0.011 mg/mL, respectively. Zhou et al.’s [23] investigation for seven varieties of wheat bran phytochemicals from different countries found a wide range of phenolic acids, tocols, and carotenoids. The ferulic acid concentration was 99−231 μg/g, while α-, δ-, and γ- tocopherols yields were up to 21.29, 7.0, and 6.90 μg/g, respectively, and lutein, zeaxanthin, and cryptoxanthin were up to 1.80, 2.19, and 0.64 μg/g, respectively. In a different study investigating the effects of pearling on wheat [24], the authors illustrated that the antioxidant capacity of both pearled grains and byproducts of *Triticum turgidum* and *Triticum aestivum* significantly decreased as the degree of pearling increased. The maximum antioxidant capacity was found in the byproducts of 10–20 percent pearling. For increasing degrees of pearling, subsequent removal of exterior layers resulted in a drop in phenolic content as well as poorer antioxidant activity levels. Endosperm diluted the antioxidant elements when the exterior layers were eliminated, including the bran and aleurone layers, the latter being the endosperm’s outermost layer.

Phytic acid is an important plant molecule that acts as a primary reservoir of phosphates in crops. Phytic acid is also involved in plant developmental and signaling processes including auxin storage and transport, phosphatidyl inositol signaling, cell wall biosynthesis, and production of stress-related molecules [25]. The storage form of phytic acid is referred to as phytate or phytin, which is mainly located in the aleurone layer of seeds. Phytic acid accumulation negatively impacts human nutrition because of its ability to chelate micronutrients such as iron, zinc, and calcium, which reduces their bioavailability and absorption [26]. An inverse relationship was observed between iron and phytic acid, as suppressing phytic acid genes, overexpression of plant and fungal phytases generate less new phytic acid and degrade phytic acid that was already synthesized, respectively. In contrast, overexpression of plant ferritin enhances storage of iron and zinc [27], while nicotinamide synthase overexpression mobilizes them from roots to seeds [28]. Some of the main bioactive phytochemicals found in wheat are shown in Table 6.

## 4. Methodology

A systematic narrative review approach with electronic databases PubMed, Scopus, Google Scholar, and preprint platform was applied. The gathered articles are from the inception of the database until 29 September 2021. Relevant literature was retrieved by screening for pharmacological or mechanistic studies relevant to wheat (*Triticum aestivum*). Studies were reviewed if they pertained to phytochemistry and pharmacology. To obtain a complete profile of the pharmacological properties of *Triticum aestivum* and not to miss any paper, several activities were screened using the following terms: “antioxidant,” “antimicrobial,” “antibacterial,” “antifungal,” “antidiabetic,” “anticancer,” “analgesic,” “hypolipidemic.”

## 5. Results and Discussion

### 5.1. Effects of Wheat on Gastrointestinal Tract

Dietary fiber is one of the significant components of wheat. Whole grain wheat contains fermentable and nonfermentable fibers, including hemicellulose, arabinoxylan, and β-glucan, lignin, and oligosaccharides [18]. Moreover, dietary fiber is known to increase the fecal bulk. A double-blinded randomized crossover trial done by De Wit et al. [45] showed that consuming extrinsic wheat fiber-enriched products increased fecal bulk more than the conventional fiber. The mechanism behind the capability of wheat fiber to enhance the fecal bulk is assumed to be caused by the insoluble components of wheat that colonic bacteria cannot degrade. Hence, it can absorb water in the colon, thereby increasing the fecal bulk [46]. Another mechanism hypothesized by McRorie and McKeown [47] is that the intestinal transit time is accelerated by the mechanical irritation as well as simultaneous stimulation of both secretions of water and mucus in the large intestines by the insoluble fiber. This effect is beneficial for constipation, especially for the elderly, as intestinal complaints are a frequent health complaint reported by geriatrics and their care providers. According to Spinzi [48], treatment results often improve the quality of life in the elderly.

The human gut microbiota is a complex and dynamic community consisting of bacteria, fungi, and viruses. Therefore, maintaining a healthy gut and regulating the host immune system is crucial. In addition, any disruptions in microbial composition have been linked to multiple metabolic disorders such as diabetes mellitus, obesity, colorectal cancer, and other diseases [49]. Many components of wheat have been reported to have protective activity against chronic superficial gastritis, as reported by Kan et al. [50], where wheat peptides and fucoidan play an essential role in altering gut microbiota and short-chain fatty acid production (SCFA), which was proved to be beneficial for the lower gastrointestinal tract [51]. SCFA is a crucial metabolite produced by bacterial species that feed on non-digestible dietary fibers known as prebiotics [52]. Prebiotics are non-digestible food ingredients that promote the growth of beneficial microorganisms in the intestines. In wheat, the natural prebiotics is fructans and arabinoxylans, non-digestible oligosaccharides; these prebiotics are known to significantly increase prebiotics in Iron (Fe)-deficient broiler chickens. In addition, results showed an increase in the relative amounts of *bifidobacteria* and *lactobacilli* in the presence of those prebiotics [53]. Another study done by Christensen et al. [54] revealed that wholegrain wheat consumption increases the abundance of *bifidobacteria* compared to baseline. In addition, *bifidobacteria* have been indicated to possess beneficial effects on intestinal homeostasis either through direct epithelial contact or through metabolic production. A study done by Müller et al. [55] on the impact of wheat bran-derived prebiotic supplements for their effects on the gastrointestinal transit showed that there is a softer stool consistency after intervention with arabinoxylan-oligosaccharides (AXOS), as well as being able to modulate gut microbiota (increase in *Bifidobacterium* and to a lesser extent *lactobacillus* among other bacteria species).

### 5.2. Effects of Wheat on Metabolic Disease

Metabolic diseases involve disruptions in normal metabolism typically due to enzyme deficiency or accumulation of enzymes or toxins arising from an endocrine organ disease, organ malfunction, inadequate intake, dietary deficiency, or malabsorption [56]. However, inherited metabolic illnesses, also known as inborn errors of metabolism, can be inherited or acquired over one’s lifetime. Inherited metabolic illnesses affect less than one in every 3000 infants, making them uncommon [57]. Diabetes mellitus is a group of metabolic disorders of carbohydrate metabolism characterized by hyperglycemia, usually resulting from insufficient insulin production (type 1 diabetes) or an ineffective response of cells to insulin (type 2 diabetes). In 2019, the global prevalence of diabetes was estimated to be 463 million people, and the numbers are expected to rise to 578 million people by 2030. Furthermore, the global prevalence of impaired glucose tolerance is estimated to be 374 million in 2019 and will reach 454 million by 2030 [58]. Diabetes mellitus has become a global challenge concerning its social, economic, and clinical burden. Type 2 diabetes (T2D) has been identified as an inflammatory disease due to inflammatory factors that are present in T2D [59]. The incidence of T2D is significantly high in overweight or obese individuals with visceral adiposity correlated with its pathological conditions: hyperglycemia, dyslipidemia, insulin resistance, and hypertension [60]. Therefore, lifestyle modifications are an integral part of managing T2D. Such modifications include promoting a moderate bodyweight reduction, increasing regular exercise and diet.

Few in vitro investigations have found that ancient Italian wheat includes many wheat species such as T.A. in addition to others; namely, Khorasan wheat (*Triticum turgidum*), einkorn (*Triticum monococcum*), emmer (*Triticum dicoccum*), and spelt (*Triticum spelta*) have antioxidant and anti-inflammatory properties [61]. In Zucker diabetic fatty (Z.D.F.) rats, the physiological effects of ancient wheat whole grain flour diets on the development and progression of type 2 diabetes and acute glycemic responses have been reported. It has been found that ancient wheat diets resulted in reducing total and LDL-cholesterol as well as the downregulation of essential regulatory genes associated with glucose and fat metabolism, which is equal to diabetes prevention or delay. Compared to wheat, spelt and rye produced a lower acute glycemic response [62]. Furthermore, several investigations compared the immunological toxicity profile of ancient wheat cultivars to current kinds for celiac disease. According to different data, ancient wheat varieties, on average, have lower numbers of immunoreactive T cells than modern wheat types. Immune responses differed significantly depending on genotype, as determined by epitope-specific T-cell responses, in studies utilizing protein extracts from ancient and modern wheat types. Spelt wheat’s cytotoxicity was comparable to that of TA [63]. Moreover, there is an equivalent reduction of cell development, activation of apoptosis, nitric oxide release, tissue transglutaminase, and modulation of transepithelial electrical resistance on Caco-2/Tc7 and K562 cell agglutination when comparing spelt with T.A. Also, T-cell activity and INF-gamma release from 4 children with celiac disease changed significantly following exposure to 9 different wheat landraces, including T.A. [64,65]. A randomized, double-blinded crossover experiment comparing the effect of replacing bread with the ancient grain variants “Verna: T.A.” with modern grain variations on cardiovascular risk profiles was carried out. Consumption of bread made from ancient types resulted in substantial improvements in several cardiovascular parameters after eight weeks of intervention. Indeed, the older varieties have been demonstrated to lower total cholesterol, low-density lipoprotein (LDL) cholesterol, and blood glucose levels. Furthermore, after consuming products derived from the ancient “Verna” type, a considerable rise in circulating endothelial progenitor cells was discovered, thus lowering cardiovascular risk factors [66].

#### 5.2.1. Antidiabetic Effect

Recent research for Müller et al. [55] has reported that a 12-week intervention study with wheat bran extract of Arabinoxylan-Oligosaccharide (AXOS) leads to a decreased postprandial glucose concentration compared to the placebo. Besides, three meta-analyses studies showed that regular whole-grain intake had been consistently associated with a lower risk of T2D [67,68,69]. Moreover, eleven prospective studies reported by Priebe et al. [69] showed consistent results of a reduced risk of T2DM for consuming a higher intake of whole grain (27% to 30%) or cereal fiber (28% to 37%). These results prove that whole-grain intake has the potential to reduce the risk of T2DM.

In a study for wheat seeds’ antidiabetic activity and mechanism, Ajiboye et al. [70] found that fasting blood glucose, albumin, globulin, bilirubin, urea, creatinine, Na, and K levels were dramatically reduced by a diet based on T.A. seeds. Furthermore, diabetic rats on a diet rich in T.A. seeds had significantly higher insulin and glycogen levels. Additionally, hexokinase, catalase, superoxide dismutase, and glutathione peroxidase levels as antioxidants were also elevated. In the same study, diabetic rats fed T.A. seed had significantly lower activities of glucose-6-phosphatase and fructose 1,6-diphosphatase, as well as a lower concentration of malondialdehyde, and reversed the activities of liver function such as alanine transferase, gamma-glutamyl transferase, alkaline phosphatase, and liver, kidney, and pancreas tissue regeneration, compared to the control group. Flavonoids have been linked to hypoglycemic effects and protection against oxidative stress due to their ability to scavenge free radicals and contain possible antioxidant qualities. Notably, wheatgrass is used for its health advantages, and wheat is an important food crop farmed worldwide. In streptozotocin-induced diabetic rats, coadministration of a polyphenol-rich wheatgrass meal “9 days after germination” has controlled hyperglycemia, glycosuria, and regained body weight [71]. Flavonoids such as apigenin have been shown to enhance liver functions in diabetic rats fed a high-fat diet.

Insulin insufficiency also inactivates lipase, which raises blood phospholipid levels. Polyphenols such as flavones and hydroxycinnamic acids inhibited adipogenesis and gluconeogenesis in various animal models by inducing glucokinase activity and thereby suppressing adipogenesis and gluconeogenesis [72]. In diabetes pathogenesis, oxidative stress causes the production of reactive oxygen species, which leads to glucose auto-oxidation, changes in antioxidant enzymes, and the generation of lipid peroxides. Damage caused by lipid peroxide impairs membrane function, increases membrane stiffness, and generates cellular deformability, all of which are typical features in the progression of diabetes. Al-Numair et al. [73] illustrated that the administration of kaempferol to diabetic rats resulted in a near-normalization of plasma glucose, insulin, lipid peroxidation products, enzymatic and non-enzymatic antioxidants. It also showed that the amount of lipid peroxidative indicators in their plasma and tissues was dramatically reduced by kaempferol. Similarly, the diabetic rats received ferulic acid supplementation, resulting in lower glucose levels, TBARS, hydroperoxides, free fatty acids, and glutathione levels. In addition, ferulic acid enhanced superoxide dismutase, catalase, and glutathione peroxidase activity and expanded pancreatic islet size [74]. In the same context, diacylglycerol (DAG) and protein kinase C (PKC) increased levels are associated with retinal and renal dysfunctions in diabetes. Therefore, Koya et al. [75] tested alpha-tocopherol on streptozotocin-induced diabetic mice to prevent glomerular hyperfiltration and albuminuria and PKC activities increase. In addition, DAG content and PKC activity increase was controlled by the effect of alpha-tocopherol on the glomeruli with significant improvement in glomerular filtration rate and filtration fraction.

#### 5.2.2. Cholesterol-Lowering Effect

In early research to study the effect of wheat bran fiber and calcium on fecal bile acids in patients with resected adenomatous colon polyps, Alberts et al. [76] hypothesized that a high intake of fiber and calcium reduced the concentrations of fecal bile acids. Fecal bile acids represent an essential pathway for eliminating cholesterol from the body. A more recent study done by Tong et al. [77] reported that wheat bran arabinoxylans reduced the total plasma- and LDL-cholesterol concentrations in hypercholesterolemic hamsters fed with a high-cholesterol diet containing 0.5% arabinoxylans. The study also reported that arabinoxylans contributed to cholesterol absorption inhibition in the intestines as well as the augmentation of bile acids excretion. This study indicates that an increased intake of arabinoxylan is one of the possible ways to prevent cardiovascular diseases.

One of the critical processes by which phytosterols decrease cholesterol in the body is by reducing cholesterol absorption. Because plant sterols and cholesterol are both water-insoluble, they must be emulsified in the presence of bile acids to produce micelles that can be absorbed by small intestinal cells. Because phytosterols have a chemical structure similar to cholesterol, they compete with cholesterol for a space in micelles, limiting cholesterol absorption by small intestine cells [78]. In addition, the transporter protein (NPC1L1) plays an essential role in cholesterol absorption in the gut. NPC1L1 transports cholesterol molecules from the small intestinal mucosal surface to the cells. After treatment with stigmasterol or sitosterol, the expression of NPC1L1 in liver cells was dramatically reduced, suggesting that phytosterol may also reduce cholesterol absorption in liver cells [79]. On top of that, sterol-regulatory element-binding proteins (SREBP) are cholesterol regulatory proteins that function as an intracellular cholesterol sensor in the endoplasmic reticulum and are capable of intracellular cholesterol feedback control. Consequently, SREBP is primarily responsible for regulating cholesterol production through activating and entering it into the nucleus when intracellular cholesterol is lower than usual, thereby triggering the expression of numerous genes involved in cholesterol production [80]. In addition, SREBP level in the nucleus and cholesterol plasma content in rat liver were considerably reduced after 28 days of feeding golden hamsters with plant sterol combination, demonstrating that phytosterol may lower cholesterol production by suppressing SREBP expression [81]. In the same context, wheat bran extract with fructans and arabinoxylans are potential prebiotic nutrients for preventing obesity and associated metabolic diseases. The induction of both *Bifidobacterium animalis* and *Bifidobacterium pseudolongum* by wheat prebiotics in the caecum demonstrated wheat bran extract’s bifidogenic action. Wheat bran extract supplementation reversed Western diet-induced fat mass expansion, steatosis, hypercholesterolemia, hyperleptinemia, hyperglycemia, and hyperinsulinemia to normal levels. The addition of wheat bran extract to Western diet-fed mice, reduced glucose-dependent insulinotropic polypeptide (GIP) release that might represent a protective mechanism against adipose tissue storage, and hepatic steatosis [82].

### 5.3. Effects of Wheat on Cancer

In both human and animal trials, wheat bran supplementation has been proven to protect against the development of various malignancies, including those of the colon and breast. Dietary fiber only accounts for around half of wheat bran that contains minerals and phytochemicals, which may help prevent cancer. Phytic acid and different phenolic components such as phenolic acids, lignans, and flavonoids are examples of these ingredients [83]. According to Aune et al. [84] and Zhang et al. [85], the correlation between high consumption of whole grains and cancer prevention is noticeable. In particular, the Australian polyp prevention project investigated people who had one or more colonic polyps removed. It was shown that adding wheat bran to people’s diets for 1–2 years lowered their likelihood of getting a second adenoma with a diameter bigger than 10 mm, especially if they were on a low-fat diet [86]. Furthermore, the effects of adding wheat bran to the diets of 62 premenopausal women were investigated. Finally, Rose et al. [87] found that wheat bran reduced blood estradiol and estrone during the follicular phase of the cycle, leading to a lower risk of breast cancer.

#### 5.3.1. Colorectal Cancer

It is estimated that 1 in 23 men and 1 in 25 women are at risk of developing colorectal cancer in their lifetime [88]. According to GLOBOCAN 2018, colon and rectal cancers are the fourth and eighth most common cancer globally, respectively [89]. It has been claimed that some components of wheat are said to reduce the risk of colorectal cancer. Zhu et al. [90] reported that a wheat fraction containing alkylresorcinols has the most potent antiproliferation effect when tested on human HCT-116 and HT-29 colon cancer cells. Another study done by Zhao et al. [3] demonstrated a breakthrough and a clear correlation of the synergistic effects of the alkylresorcinol C21 with the intestinal microbial butyrate, where the combination resulted in the antiproliferative effects on HCT-116 and HT-29 cells. Another study done by Qu et al. [91] found that lignan metabolites of wheat bran significantly inhibited SW480 cell growth (colon cancer cell line). Alkylresorcinol derivatives displayed selective binding affinities towards the estrogen receptors, ERα or Erβ, in exerting the anticancer effect [92]. These studies conclude that alkylresorcinols and lignin do have anticancer effects.

Wheat aleurone is high in dietary fibers, which are fermented by the microflora and result in the synthesis of short-chain fatty acids (SCFA), known for their chemopreventive properties. Using two human colon cell lines (LT97 and HT29), Borowicki et al.’s [79] study looked at the effects of fermented aleurone on proliferation, apoptosis, differentiation, and apoptosis-related gene expression; WNT2B and p21, that were induced by the fermented supernatant. This work adds to our understanding of how wheat aleurone fermentation products can help secondary chemoprevention. Because adenoma cells were shown to be more sensitive than malignant ones, this might have significant implications for chemoprevention in the in vivo setting and eventually decreasing carcinogenesis. Interestingly, in a clinical study involving colorectal cancer patients [80], fermented wheat germ extract was effective in treating colorectal cancer in humans when used as part of supportive therapy. After a radical operation, 30 patients were given standard postoperative therapy, and 12 of them were given fermented wheat germ extract as an additive treatment. After a 9-month administration, no new distant metastases were found, compared to 4 out of 18 patients who received standard therapy alone. Seventeen patients administered fermented wheat germ extract after major surgery and chemotherapy had a better survival rate than 34 individuals. One hundred seventy colorectal cancer patients were treated with anticancer medicines (chemo/radiotherapy) in a controlled multicenter open-label cohort study, with 66 of them receiving fermented wheat germ extract. The results of this study showed that the new recurrences in the fermented wheat germ extract vs. control were 3.0% vs. 17.3%; new metastases: 7.6% vs. 23.1%; deaths: 12.1% vs. 31.7%; progression-related events in total: 16.7% vs. 42.3%. In terms of progression-free (*p* = 0.0184) and overall survival probability (*p* = 0.0278), the fermented wheat germ extract group exhibited substantial benefits.

#### 5.3.2. Breast Cancer

According to GLOBOCAN 2020, female breast cancer is the most commonly diagnosed cancer, with around 11.7% of all cancers (2,261,419 individuals) [93]. Dietary fiber has been proposed to result in the reduction of mitogenic estrogen absorption. The short- and long-term effects of dietary fiber administration revealed that rats given the high fiber diet eliminated a significant quantity of labeled estrogen, but rats on the normal or low-fiber diets excreted about half that amount over the same period. This excretion might lead to lower estrogen levels in the blood or tissue and less estrogen exposure in estrogen-sensitive tissue, subsequently lowering the chance of breast cancer development [94]. McCann et al. [95] found that dietary lignan intake causes a reduction in breast cancer mortality. On the other hand, in the meta-analysis study, Xiao et al. [96] found an association between intermediate and high intake levels of whole grain and a modest reduction in the risk of breast cancer.

Alkylresorcinols, found in whole grains, are likely useful in cancer prevention. In vitro investigations revealed that micromolar alkylresorcinols inhibited human breast cancer cell line; MCF-7 as the IC_50_ values range from 28.6 to 37.1 µM [97,98]. Importantly, the separated alkylresorcinols had an inhibitory impact on the breast cancer cell line examined that was at least 800% greater than the routinely used chemotherapy, adriamycin. In another study to examine the mechanism of alkylresorcinols [99], it has been found that alkylrespocinols suppressed the expression of CYP21A2, HSD3B2, and CYP19A1 genes, while increased the expression of CYP11A1. The impacts on steroidogenesis could not be explained by gene expression data, which might be attributable to direct effects on enzyme activities, such as suppression of CYP17A1. These findings show that alkylresorcinols decrease testosterone and estradiol production, implying a unique mechanism for alkylresorcinols in breast cancer chemoprevention. Other ingredients of T.A., such as phenolic acids, lutein, and alpha-tocopherol, have already been proven to exert their anti-breast cancer activities [100,101,102].

### 5.4. Effects of Wheat on Skin Complexion and Atopic Dermatitis

It has been known that wheat bran extract is becoming a common ingredient in skincare. Bavarsad et al. [103] investigated the hyperpigmentation disorder associated with melasma. They demonstrated the capability of a formulation containing 3.45% wheat bran extract and 0.05% tomato lycopene to reduce melasma size significantly from 6.59 ± 3.47 to 5.97 ± 3.83 with no recurrence observed one month after the end of the treatment. Furthermore, Boisnic et al. [104] elucidated wheat extract oil containing polar lipids, the antiaging properties during a placebo-controlled clinical study on 64 healthy women. According to study results, the Lemperle score in the wheat extract oil group was significantly lower than in the placebo group, reaching a clinically significant 1 grade at W12. Furthermore, the wheat extract oil group’s facial hydration improved significantly, whereas leg hydration improved after four weeks and remained throughout the supplementation period. Compared to the placebo group, skin roughness and radiance improved significantly from W0 to W8 in the wheat extract oil group. When UV-irradiated skin explants were treated with wheat extract oil, the collagen concentration was more remarkable than untreated.

Wheat sprout extract also has a significant role in treating atopic dermatitis. The anti-atopic dermatitis properties of a 70% ethanol extract were examined on both; atopic dermatitis-like skin lesions mice induced by 2,4-dinitrochlorobenzene and on TNF- and interferon-stimulated human keratinocytes in HaCaT cells [105]. Oral treatment of 200 mg/kg of the extract was administered to the mice for ten days and resulted in reducing skin thickness, transepidermal water loss, and serum immunoglobulin E levels. Wheat sprout extract also inhibited the release of inflammatory chemokines in TNF and IFN-stimulated HaCaT cells by regulating the signal transducer and activator of transcription-1 and suppressing cytokine signaling pathways.

### 5.5. Antimicrobial Effects of Wheat Extracts

Jeong et al.’s [106] study aimed to assess the antibacterial properties of three wheat sprout extracts; water, ethanol, or methanol. Results showed that water-derived phenolic compounds from wheat sprouts inhibited the development of oral microorganisms *Actinomyces viscosus*, *Streptococcus mutans*, and *Streptococcus salivarius*. According to paper-disc agar-diffusion studies, phenolic compounds, particularly ferulic and sinapic acids, had the most potent antibacterial activity. In the same context, 1 mg/mL water extract of seven-day-old fresh wheatgrass was found bacteriostatic/bactericidal against two Gram-positive, two Gram-negative bacteria and one fungus foodborne microorganisms [107]. *Bacillus cereus*, followed by *Staphylococcus aureus*, were found to be the most sensitive, while Gram-negative *Escherichia coli* was the least sensitive. Moreover, the hexane extract of wheatgrass rich in carotene and chlorophyll, 0.54 ± 0.016 g/L and 0.42 ± 0.066 g/L, respectively, exhibited potential effects against *Salmonella Enteritidis* with inhibitory zones in the range of 9–8 mm along with a minimum inhibitory concentration (50 mg/mL) and reduction of cell count [108].

Alkylresorcinol derivatives were also found to exhibit antibacterial activity; thus, they were used to treat infections. 4-hexylresorcinol also exerted bacteriostatic action on seven different phytopathological bacterial strains. These aliphatic derivatives of resorcinols have been highly active against Gram-positive bacteria, particularly acid-resistant *Mycobacterium smegmatis* and *Mycobacterium tuberculosis*. Also, in a clinical trial with over 200 patients, a combination of monounsaturated alkylresorcinols and their monomethyl derivatives showed high efficacy of >80% of patients in tuberculosis therapy. This topic was reviewed extensively by Kozubek and Tyman [109]. As one of the components of wheat grains, lignans also exhibit a potent antibacterial, antifungal, and antiviral effects. For instance, the lignan derivative, Nortrachelogenin, displays a robust and diversified antibacterial action against various bacteria, including antibiotic-resistant strains (MIC = 2.5 or 5.0 g/mL). In mechanistic research, this compound exerted its activity by damaging the bacterial membrane of *Escherichia coli*. In addition, Hinokinin was found to have antibacterial properties against *S. aureus* (MIC = 0.0458–3.125 mg/mL) and methicillin-resistant *staphylococcus aureus* isolates [110].

### 5.6. Anti-Inflammatory Effects of Wheat Extracts

Choi et al. [111] studied the anti-inflammatory properties of 80% ethanolic wheatgrass extract fractionated into ethyl acetate and n-butanol fractions. The HPLC analysis elucidated the presence of benzoic acid, quercetin, and luteolin abundantly. Those hydrophobic fractions inhibited the expression of inducible nitric oxide synthase gene, nuclear factor-kappa B (NF-κB) and reduced phosphorylation of MAPK. The RAW 264.7 cell line and animal model were also used to investigate this process induced by polyphenolic components of T.A. Differently, four different flavolignans and one flavone were extracted from the wheat hull and connected to LPS-induced nitric oxide production inhibition in RAW 264.7 cells with IC_50_ values ranging from 24.14 to 58.95 μM [112]. The hepatoprotective and anti-inflammatory effects of ethanolic T.A. sprout extract were studied by Lim et al. [113]. γ-Aminobutyric acid and α-linolenic acid contents were characterized at 209.23 and 170.92 µg/mg extract. In this study, TNF-, IL-6, and IL-1 serum and mRNA levels were dramatically elevated by acetaminophen injection, but sprout extract pre-treatment significantly downregulated them. Table 7 summarizes some of the proposed health benefits of components present in wheat grain. In addition, some components have been claimed to have ‘“antioxidant” properties.

## 6. Conclusions

In conclusion, this review summarizes the established health benefits of wheat components in various aspects. In addition, it shows the versatility and importance of wheat in human health and cosmetics. The diversity of wheat components such as dietary fibers, polyphenolic compounds, alkylresorcinols, in addition to the varying cultivars and variants, could be a key factor for this cereal grain’s biological and pharmacological activities. Moreover, the antioxidant groups such as tocopherols, tocotrienols, and carotenoids, as well as the phenolic acids, contribute to the antioxidant capacity of wheat, and consequently, they may have a critical role in chemoprevention. More clinical trials, in vivo and in vitro studies are warranted to broaden the knowledge about T.A., prevent nutrition-related diseases, and increase consumers’ physical and mental well-being.

## Figures and Tables

**Table 1:** Parts of wheat bran, definition and function of each part.

Part	Definition	Function	Ref.
Wheat bran	Protective outermost shell of the wheat kernel that is stripped away during the milling process.	Protects inner layer of the grain from external weather, insect, molds, and other microorganisms’ attack.	[10]
Wheat grain or whole grain	The grain portion of the wheat plant and the source of flour. Contains endosperm, germ, and bran.	Proteins and other nutrients storage	[11]
Wheat germ	Contains plant embryo	Responsible for plant reproduction	[12]

**Table 2:** Taxonomical hierarchy of T.A. Adopted from www.itis.gov (accessed on 22 August 2021).

Kingdom	Plantae
Subkingdom	Viridiplantae
Infrakingdom	Streptophyta
Superdivision	Embryophyta
Division	Tracheophyta
Subdivision	Spermatophytina
Class	Magnoliopsida
Superorder	Lilianae
Order	Poales
Family	Poaceae
Genus	*Triticum* L. wheat
Species	*Triticum aestivum* L.
Synonyms	*Triticum hybernum* L., *Triticum sativum* Lam., *Triticum vulgare* Vill.

**Table 3:** General nutrient composition of wheat bran.

Bran Component	Percentage of the Dry Matter	Reference
Dietary fiber	33.4–63.0	[15]
Moisture	8.1–12.7	[15]
Ash	5.7–6.5	[15]
Protein	9.60–18.6	[15,16]
Total Carbohydrates	60.0–75.0	[17]
Starch	9.10–38.9	[15,16]

**Table 4:** Micronutrients found in wheat bran.

Micronutrients	mg per 100 g	Reference
Phosphorus	900–1500	[18,19]
Magnesium	530–1030	[19]
Zinc	8.3–14.0	[19]
Iron	1.9–34.0	[18,19]
Manganese	0.9–10.1	[18,19]
Vitamin E (tocopherols/tocotrienol)	0.13–9.5	[18]
B vitamins		
Thiamin (B1)	0.51–1.6	[18]
Riboflavin(B2)	0.20–0.80	[18,19]
Pyridoxine(B6)	0.30–1.30	[19]
Folate(B9)	0.088–0.80	[18]

**Table 5:** Nutrient composition in whole wheat grain flour.

Nutrient	Unit	Value per 100 g
PROXIMATES		
Water	g	10.74
Energy	kcal	340
Protein	g	13.21
Total lipid (fat)	g	2.50
Carbohydrate,	g	71.97
Fiber, total dietary	g	10.7
Sugars, total	g	0.41
MINERALS		
Calcium, Ca	mg	34
Iron, Fe	mg	3.60
Magnesium, Mg	mg	137
Phosphorus, P	mg	357
Potassium, K	mg	363
Sodium, Na	mg	2
Zinc, Zn	mg	2.60

Source: USDA Nutrient Database.

**Table 6:** Classification of bioactive phytochemicals present in T.A.

Classification	Phytochemical Group	Percentage	Distribution in the Plant Part	Example	Structure	Ref.
Phenolic acids	Hydroxy benzoic acid or hydroxycinnamic acid derivatives	Bran (6.85 mg/g D.M.) Aleurone (10 mg/g D.M.) Pericarp (14.56 mg/g D.M.)	Phenolic acids are rare in the endosperm, abundant in germ and bran	Ferulic acid	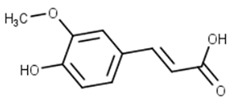	[29,30,31]
Tocopherols	Tocopherols and tocotrienols	Wheat germ oil (273 mg/100 g) wheat bran oil (190 mg/100 g)	α-tocopherol is the predominant tocol in leaves, γ-tocopherol in other plant parts, whereas the distribution of tocotrienols is variable.	Alpha-tocopherol	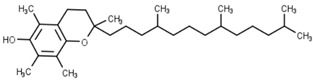	[32,33]
Carotenoids	Carotenoids	Wheat germ oil (12.23 mg/100 g) wheat bran oil (2.21 mg/100 g)	Carotenoids are abundant in the germ but are more plentiful in the endosperm fraction, which contributes 72% of total kernel lutein	Lutein	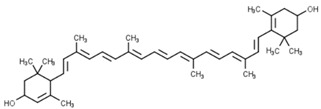	[32,34]
Phenolic lipids	Alkylresorcinols	2672–3645 μg/g	99% alkylresorcinol content was detected in the caryopsis’ intermediate layer, which included the hyaline, testa, and inner pericarp, and nothing was identified in the endosperm or germ.	5-Heptadecylresorcinol	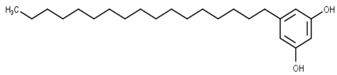	[35,36]
Miscellaneous groups	Benzoxazinoids	Wheat flour 1648 ng/g Wheat germ 121,221 ng/g D.M.	High concentration in roots could occur at later stages of plant development; however, in the seedling stage, it shows a much higher root/foliar concentration ratio	2, 4-dihydroxy-1,4-benzoxazin-3-one	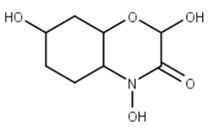	[37,38]
	Lignans	483–1515 μg/100 g	Lignans are predominant in the aleurone layer	Secoisolariciresinol	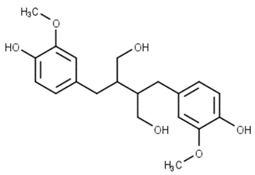	[39,40]
	Phytosterols	Up to 2117 μg/g	Phytosterols are accumulated in the bran and germ of wheat, and they are more evenly distributed in the intermediate layers and aleurone cell contents	Sitosterol	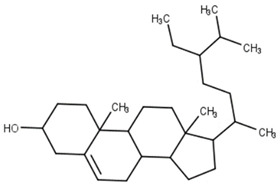	[41]
	Steryl ferulates (Known also as γ-oryzanols, are a mixture of ferulic acid esters of sterols and triterpene alcohols)	6.3–29 mg/100 gm	Steryl ferulates are accumulated in the intermediate of bran and germ of the wheat.	Campesteryl ferulate	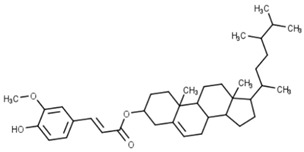	[13,41,42]
	Free fatty acids	2.5–3.5%	In bran, germ and endosperm	α-linolenic acid	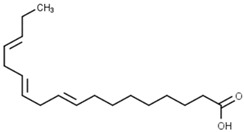	[13,43]
	Oxylipins	-	In bran, germ and endosperm	13-Hydroxyoctadecadienoic acid (13-HODE)	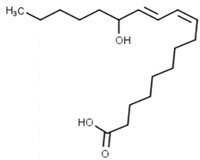	[13]
	Galactolipids	4049–4627 nmol/g of sample for the whole wheat flour in Overley and Alpowa cultivars	In the endosperm	Monogalactosyl-diacylglycerol	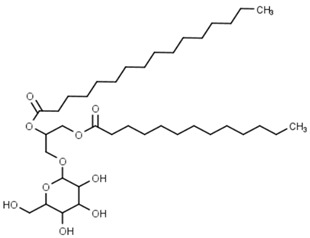	[13,44]
	Phospholipids	713–1080 nmol/g of sample for the whole wheat flour in Overley and Alpowa cultivars	In the endosperm	Phosphatidylcholine 18:1–18:2	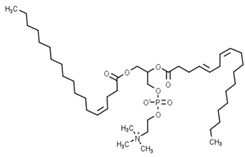	[13]

**Table 7:** Summary of proposed health benefits and their health claims.

Component of Wheat	Proposed Health Benefits	Supported by Approved Health Claims ^a^
Dietary fiber	Increase in fecal bulk Reduction in intestinal transit time Contribution to the maintenance or achievement of a normal body weight	Yes
	Reduce the risk of colorectal and breast cancer Reduce the risk of stroke Prebiotic effects Stimulation of immune responses	No
Resistant starch	Reduce postprandial glycemic response	Yes
Phenolic acids	Vascular function improvements Antitumor properties	No
Alkylresorcinols	Antimicrobial properties Anticancer properties	No
Lignans	Phytoestrogen properties Anticancer properties Antimicrobial properties	No
Tocols	Vitamin E activity	Yes
	Prevention of neurodegeneration Induction of immune responses Anticancer effects Reduction of cholesterol Antioxidant effects	No

^a^ Approved by the European Food Safety Authority (EFSA).

## Data Availability

Not applicable.
